# Haploidentical hematopoietic stem cell transplantation compared to immunosuppressive therapy plus eltrombopag as first-line treatment for severe aplastic anemia patients aged 40–65: a multicenter retrospective study

**DOI:** 10.1007/s00277-026-07289-2

**Published:** 2026-09-29

**Authors:** Xiaohui Xiao, Guiping Chen, Cong Xu, Liangliang Wu, Xiaoyan Liu, Ming Zhou, Xiaowei Chen, Ruiqing Zhou, Wenjian Mo, Yumiao Li, Shilin Xu, Yuping Zhang, Dongjun Lin, Shunqing Wang

**Affiliations:** 1 Department of Hematology, The Seventh Affiliated Hospital, Sun Yat-Sen University, Shenzhen, China; 2 Department of Hematology, Guangzhou First People’s Hospital, Institute of Blood Transfusion and Hematology, Guangzhou Medical University, Guangzhou, China; 3 Department of Hematology, Guangzhou First People’s Hospital, South China University of Technology, Guangzhou, China; 4 Department of Hematology, Sun Yat-sen Memorial Hospital, Sun Yat-sen University, Guangzhou, China; 5 Guangdong Provincial Key Laboratory of Malignant Tumor Epigenetics and Gene Regulation, Sun Yat-Sen Memorial Hospital, Sun Yat-Sen University, Guangzhou, China

**Keywords:** Haploidentical hematopoietic stem cell transplantation, Immunosuppressive therapy, Severe aplastic anemia

## Abstract

Immunosuppressive therapy (IST) plus eltrombopag (EPAG) (IST+EPAG) is the preferred first-line treatment for severe aplastic anemia (SAA) patients over 40 years. However, direct comparison between IST+EPAG and upfront haploidentical hematopoietic stem cell transplantation (HID-HSCT) in this population remains limited. We retrospectively analyzed 123 newly diagnosed SAA patients aged 40–65 who received first-line HID-HSCT (*n* = 67) or IST+EPAG (*n* = 56) between 2016 and 2024. Hematological response, survival, and safety were compared. The HID-HSCT group demonstrated superior complete response (CR) rates at 3 months (17.9% vs. 5.4%), 6 months (44.8% vs. 17.9%), and 12 months (64.2% vs. 33.9%) (all *P* < 0.001). The 3-year overall survival (OS) was comparable between the HID-HSCT and IST+EPAG groups (78.0% vs. 80.3%, *P* = 0.662), and a landmark analysis conditional on survival to 6 months confirmed comparable subsequent 3-year OS (92.2% vs. 88.9%, *P* = 0.616). Meanwhile, the 3-year failure-free survival (FFS) was significantly higher in the HID-HSCT group among patients aged 40–49 (82.9% vs. 60.1%, *P* = 0.045), whereas no difference was observed in those aged 50–65 (*P* = 0.984). HID-HSCT may offer more durable benefits in selected SAA patients aged 40–49, while its advantage over IST+EPAG is not evident in patients aged 50–65.

**Supplementary Information:**

The online version contains supplementary material available at https://doi.org/10.1007/s00277-026-07289-2.

## Introduction

 Severe aplastic anemia (SAA) is commonly treated with either hematopoietic stem cell transplantation (HSCT) or immunosuppressive therapy (IST) combined with eltrombopag (EPAG) [1–3]. For younger adults, HSCT is recommended as the preferred treatment for patients with SAA [4], but it is often avoided in patients aged 50 and older due to concerns about complications and treatment-related mortality [5]. For patients over 40 years of age, particularly those without a matched sibling donor (MSD), IST in combination with EPAG (IST+EPAG) is a standard first-line treatment [6, 7]. However, relapse occurs in 30% to 60% of patients receiving IST, which typically consists of anti-thymocyte globulin (ATG) and cyclosporin A (CsA) and years of prolonged CsA therapy are required [3, 8]. EPAG, an oral small-molecule peptide TPO mimetic, has demonstrated improved complete response (CR) rates in the randomized controlled RACE trial [2]. The study of Danielle M. et al. reported a 6-month CR increase from 10% to 58% when EPAG was added to standard IST [9]. Despite the improvement of EPAG, issues such as non-response, relapse, and clonal evolution remain challenging in older patients [10, 11]. Overall survival (OS) following IST is age dependent [12], and treatment decisions for older patients should be carefully assessed whether they are fit enough to receive ATG or not [13]. 

With advances in HSCT leading to improved safety and broader donor availability, haploidentical HSCT (HID-HSCT) has emerged as a complementary treatment for patients lacking an MSD or a matched unrelated donor (MUD). This progress has ushered in a new era in which “everyone has a donor.” [14–16] Data from the Chinese Blood and Marrow Transplant Registry reveal that HID-HSCT is not only increasingly utilized in SAA patients [17], but also the upper limit of age for transplantation in SAA patients has been steadily increased [5, 12, 18–20]. Our previous studies have demonstrated that for SAA patients aged 40 years or older, HID-HSCT is a feasible alternative treatment option, with estimated 3-year OS rates comparable to those in the MSD and MUD groups [20]. Liu et al. reported that, compared with first-line IST plus EPAG, first-line HID-HSCT accelerated hematopoietic recovery and led to superior failure-free survival (FFS) and health-related quality of life (HRQoL) in patients under 40 with very severe aplastic anemia (VSAA) and in those aged 40–55 with SAA [21]. 

However, data directly comparing first-line HID-HSCT with IST plus EPAG in SAA patients over 40 are lacking, particularly from large patient cohorts. We enrolled 123 newly diagnosed SAA patients aged 40–65 years: 56 received IST+EPAG and 67 underwent HID-HSCT. This study aims to evaluate the safety and efficacy of HID-HSCT as a first-line treatment in this older population, thereby addressing a critical gap in the evidence and assessing its viability.

## Materials and methods

### Patients with SAA

This study enrolled 123 consecutive patients with newly diagnosed SAA between 2016 and 2024, from Guangzhou First People’s Hospital, Sun Yat-sen Memorial Hospital, and the Seventh Affiliated Hospital, Sun Yat-Sen University. Inclusion criteria were: (1) age 40–65 years, (2) meeting the standard diagnostic criteria for SAA [3], and (3) receiving first-line treatment with either IST plus EPAG or HID-HSCT because of the absence of an MSD. The exclusion criteria were: (1) congenital bone marrow disorders such as Fanconi anemia, Diamond-Blackfan anemia, and dyskeratosis congenita (DKC) proven by clinical analysis and laboratory assays; (2) myelodysplastic syndrome (MDS) based on morphology, flow cytometry, and karyotype according to the World Health Organization (WHO) 2016 edition criteria; (3) pregnancy or lactation. Very severe aplastic anemia (VSAA) was defined as an absolute neutrophil count (ANC) of < 0.2 × 10^9^/L in patients otherwise meeting SAA criteria. According to current guidelines, IST+EPAG or alternative donor transplantation is recommended as first-line treatment for SAA patients lacking an MSD, with the final decision based on donor availability, disease severity, and patient preference. Of the 123 patients enrolled, 56 received IST+EPAG and 67 underwent HID-HSCT. Written informed consent was obtained, and the study was approved by the Ethics Committee of Guangzhou First People’s Hospital. The last follow-up date was January 2026.

### IST+EPAG procedure

Patients in the IST+EPAG group received rabbit ATG at a dose of 3.5 mg/kg/day intravenously for 5 consecutive days. Oral CsA was initiated at 5 mg/kg/day on day 1, with subsequent dose adjustments to maintain a trough concentration of 150–200 ng/mL based on clinical response and adverse effects. CsA was continued for at least 6 months. EPAG was administered at a dose of 75–150 mg/day for at least 3 months.

### HID-HSCT procedure

Patients in the HID-HSCT group received conditioning with either fludarabine/cyclophosphamide (Flu/Cy)-based or busulfan/cyclophosphamide (Bu/Cy)-based protocols. The specific regimens included a standard BuCy protocol and modified post-transplant cyclophosphamide (PTCy) protocols, detailed as follows: Bu at 3.2 mg/kg/day intravenously (i.v.) for 2 days, Cy at 40 mg/kg/day i.v. on days − 5 to − 2, ATG at 2.5 mg/kg/day i.v. for 4 days (BuCy); Flu at 30 mg/m^2^/day i.v. for 4 days, Cy: 40 mg/kg/day i.v. on days − 6, −5, + 3, and + 4, Bu: 3.2 mg/kg/day i.v. for 2 days, ATG: 2 mg/kg/day i.v. for 3 days (PTCy-Bu); Flu: 30 mg/m^2^/day i.v. for 4 days, Cy: 40 mg/kg/day i.v. on days − 6, −5, + 3, and + 4, ATG: 2 mg/kg/day i.v. for 3 days, total body irradiation (TBI) 4 Gy on day − 1 (PTCy-TBI).

The target doses were ≥ 6 × 10^8^/kg for mononuclear cells and ≥ 2 × 10^6^/kg for CD34 + cells. Fresh and unmanipulated bone marrow or peripheral blood stem cells (PBSC) were infused on the day of their collection. For modified PTCy regimens, graft versus host disease (GVHD) prophylaxis consisted of PTCy (40 mg/kg on days + 3 and + 4), CsA (target trough 200–400 ng/mL), and mycophenolate mofetil (MMF) (0.5 g, q12h). For the BuCy regimen, CsA combined with MMF and short-term methotrexate (MTX) was given as described previously [22, 23]. Acute GVHD (aGVHD) and chronic GVHD (cGVHD) were evaluated according to international criteria [24]. Granulocyte colony-stimulating factor (G-CSF) was administered starting day + 5 until ANC > 1.0 × 10⁹/L for 3 consecutive days. All HID-HSCT patients received posaconazole from day − 7 until engraftment and for 100 days in patients with prolonged neutropenia or GVHD on steroids. Trimethoprim-sulfamethoxazole was given for Pneumocystis jirovecii pneumonia prophylaxis until at least 6 months post-transplant, or longer in patients receiving ongoing immunosuppressive therapy. Ganciclovir (5 mg/kg twice daily) was given intravenously from day − 8 to day − 1 pre-transplantation. Starting on day + 7 post-transplantation, letermovir was administered as CMV prophylaxis in patients who received transplantation after 2023 (*n* = 16), when letermovir became clinically available in China.

### Definitions and evaluations

Complete response (CR) was defined as ANC > 1.5 × 10^9^/L, platelet count > 100 × 10^9^/L, and hemoglobin > 100 g/L. Partial response (PR) was defined as transfusion independence and no longer meeting SAA diagnostic criteria [3]. No response (NR) was defined as blood counts still meeting severe disease criteria [3]. Overall response rate (ORR) encompassed CR and PR. OS was calculated from the start of treatment (ATG for IST+EPAG group or transplantation for HID-HSCT group) to death or last follow-up. FFS in the IST+EPAG group included non-response at 6 months, relapse, clonal evolution, secondary malignancies, disease progression requiring intervention, and death. FFS in the HID-HSCT group was calculated from stem cell infusion to graft failure, relapse, secondary malignancies, or death. GVHD-free failure-free survival (GFFS) was defined as survival without grade III-IV aGVHD, moderate-severe cGVHD, or treatment failure as described above. Performance status was graded according to Eastern Cooperative Oncology Group (ECOG) scoring before treatment. All transplant-associated thrombotic microangiopathy (TA-TMA) cases were diagnosed using Jodele criteria [25]. HLA typing was performed using next-generation sequencing (NGS) methods. Typed loci included HLA-A, -B, -C, -DRB1, and -DQB1. High-resolution genotyping was achieved at the allele level. Donor-recipient matching grades were determined based on allele-level assignment.

### Statistical analysis

Data analyses were performed using GraphPad Prism 8 and R version 4.3.1 (R Foundation for Statistical Computing, Vienna, Austria). Group comparisons were performed using t-test or Mann-Whitney U test for continuous variables and chi-squared test for categorical variables. The Kaplan-Meier method was used to estimate OS and FFS probabilities, with comparisons made using the log-rank test. Cumulative incidence of aGVHD and cGVHD was estimated using the Fine-Gray competing-risk model, with death as the competing event. Multivariable Cox regression was performed adjusting for treatment group, age, and disease severity (VSAA vs. SAA), selected as the most clinically relevant covariates. Statistical significance was set at *P* < 0.05.

## Result

### Patient characteristics

**Fig. 1:**
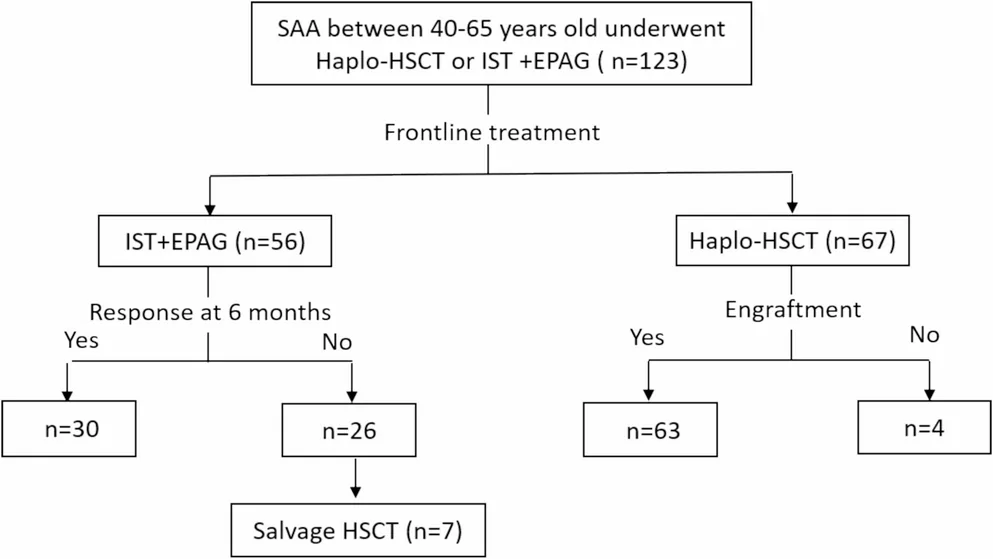
Patient selection flowchart and study design

From 2016 to 2024, 123 SAA patients (aged 40–65 years) who underwent HID-HSCT (*n* = 67) or IST+EPAG (*n* = 56) treatment were enrolled from a multicenter database (Fig. 1). Baseline demographic and clinical characteristics were well-matched between the two groups, as detailed in Table 1. The median age was 47 years in both groups, with no statistically significant difference (*P* = 0.302). Age distribution (40–49 vs. 50–65 years) and gender were also similar between the groups (*P* = 0.275 and *P* = 0.139, respectively). ECOG performance status (0–1 vs. 2–3) was comparable between the two groups (*P* = 0.909). The median time from diagnosis to treatment was 2.40 months in both groups (*P* = 0.344). The median follow-up time for alive patients was similar (*P* = 0.235). Specifically, disease severity, hemoglobin levels, platelet counts, and neutrophil counts were comparable between the groups (all *P* > 0.05).

**Table 1:** Clinical characteristics of patients in the IST+EPAG and HID-HSCT groups

	IST+EPAG *n* = 56 (%/range)	HID-HSCT *n* = 67 (%/range)	*P*
Median age	47(40–65)	47(40–60)	0.302
Age (years)			0.275
40–49	31(55.4)	48(71.6)	
50–65	25(44.6)	19(28.4)	
Gender			0.139
Male	20(35.7)	30(44.8)	
Female	36(64.3)	37(55.2)	
ECOG score			0.909
0–1	29(51.8)	34(50.7)	
2–3	27(48.2)	33(49.3)	
Disease severity			0.127
SAA	41(73.2)	39(58.2)	
VSAA	15(26.8)	28(41.8)	
With PNH clone	4(7.1)	11(16.4)	0.471
Median time between diagnosis and treatment (months)	2.40(0.27–27.87)	2.40(0.40-36.83)	0.344
Median follow-ups of alive patients (months)	53.53(0.10-110.27)	54.20(0.40-104.63)	0.235
Median hemoglobin (g/L)	64.50(30–113)	64.00(16–138)	0.594
Median platelet (×10^9^/L)	7.50(1–44)	10.00(1–68)	0.275
Median neutrophil (×10^9^/L)	0.42(0.01–1.09)	0.23(0.01-2.00)	0.161

* P < 0.05

*ECOG* Eastern Cooperative Oncology Group, *VSAA* very severe aplastic anemia, *SAA* severe aplastic anemia, *PNH* Paroxysmal nocturnal hemoglobinuria

#### Outcomes of HID-HSCT group

Key characteristics and outcomes for the HID-HSCT group were summarized in Table 2. Most patients (94.0%) received a combined bone marrow and peripheral blood stem cell graft; the remainder received peripheral blood only. No donor-recipient pairs were fully HLA-matched, with 5/10 HLA-matched pairs being most prevalent (64.2%). The distribution of donor/recipient CMV serostatus was as follows: D+/R+ (*n* = 50), D-/R+ (*n* = 9), D+/R- (*n* = 4), and D-/R- (*n* = 4). Conditioning regimens included BuCy (23.9%), PTCy-TBI (38.8%), and PTCy-Bu (37.3%). The median infused cell doses were 9.90 × 10⁸/kg for mononuclear cells and 4.56 × 10⁶/kg for CD34^+^ cells. Engraftment was rapid, with a median time of 13 days for both neutrophil and platelet recovery, and high success rates (97.0% neutrophil engraftment and 86.6% platelet engraftment by day 28).

**Table 2:** Features and outcomes of the HID-HSCT group

Transplants	*n* = 67 (%/range)
BM+PBSC	63(94.0)
PBSC	4(6.0)
ABO match	
Matched	36(53.7)
Minor mismatched	7(10.4)
Major mismatched	21(31.3)
Different	3(4.5)
CMV donor/recipient serostatus	
D+/R+	50(74.6)
D-/R+	9(13.4)
D+/R-	4(6.0)
D-/R-	4(6.0)
HLA site	
5/10	43(64.2)
6/10	13(19.4)
7/10	7(10.4)
8/10	2(3.0)
9/10	2(3.0)
Conditioning regimen	
BuCy	16(23.9)
PTCy-TBI	26(38.8)
PTCy-Bu	25(37.3)
HCT-CI score	
0	32(47.8)
1	27(40.3)
2	7(10.4)
3	1(1.5)
Median MNCs, ×10^8^/kg	9.90(7.03–18.69)
Median CD34 + cells, ×10^6^/kg	4.56(1.28–14.55)
Neutrophil engraftment, days	13(9–27)
Platelet engraftment, days	13(8-124)
28-day neutrophil engraftment	65(97.0)
28-day platelet engraftment	58(86.6)

*HLA* human leukocyte antigen, *MNC* mononuclear cell, *BM* bone marrow, *PBSC* peripheral blood stem cells, *HCT-CI* Hematopoietic cell transplantation comorbidity index, *D* donor, *R* recipient

##### GVHD in HID-HSCT group

**Fig. 2:**
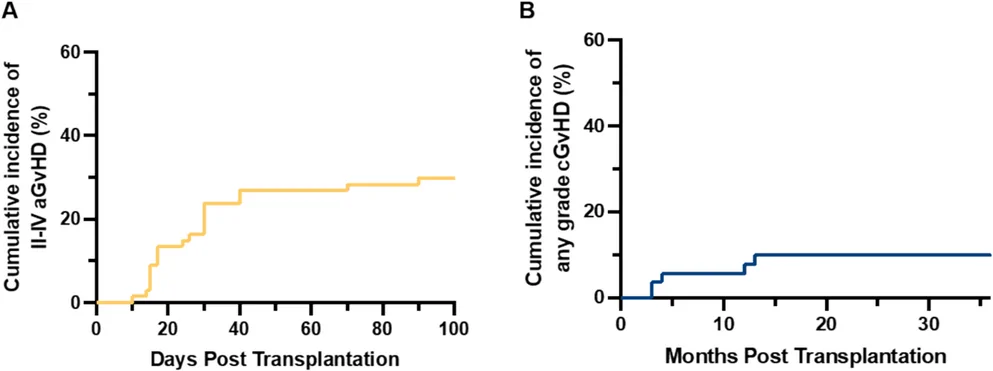
Cumulative incidence of graft versus host disease (GVHD) (**A**) The cumulative incidence of II-IV aGVHD at 100 days was 30.9% (95% CI 20.6%–43.3%); and (**B**) the 3-year cumulative incidence of cGVHD was 8.1% (95% CI 1.2%–15.1%)

The cumulative incidence of II-IV aGVHD at 100 days was 30.9% (95% CI 20.6%–43.3%, Fig. 2A); and the 3-year cumulative incidence of cGVHD was 8.1% (95% CI 1.2%–15.1%, Fig. 2B). Among the 53 HID-HSCT patients alive at last follow-up (median 54.2 months), 50 patients (94.3%) were off all systemic immunosuppression, indicating durable treatment success without ongoing GVHD-directed therapy. Overall, the HID-HSCT cohort demonstrated favorable and rapid engraftment outcomes with acceptable GVHD rates.

#### Hematological response

**Fig. 3:**
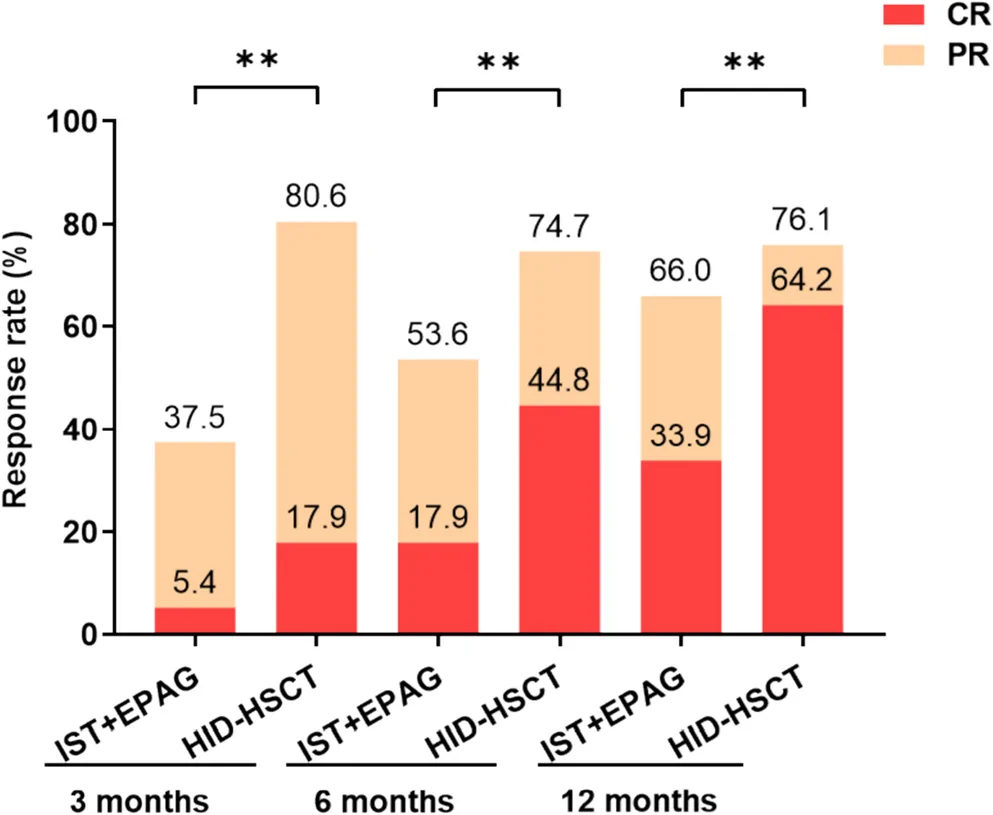
Superior hematological response in the HID-HSCT group compared to the IST+EPAG group at 3, 6, and 12 months. ** *P* < 0.01

Achieving hematological response and subsequent transfusion independence is a primary goal in SAA treatment, as it is strongly associated with improved quality of life, prolonged survival, and reduced socioeconomic burden [22, 26]. As detailed in Table 3, the HID-HSCT group demonstrated superior hematological response compared with the IST+EPAG group at all time points (*P* < 0.001 at 3, 6, and 12 months). At 3 months, the ORR was 80.6% in the HID-HSCT group versus 37.5% in the IST+EPAG group (*P* < 0.001; Fig. 3). This superior response persisted at 6 months, with ORRs of 74.7% and 53.6%, respectively (*P* < 0.001). By 12 months, the CR rate was significantly higher in the HID-HSCT group (64.2% vs. 33.9%, *P* = 0.007), as was the ORR (76.1% vs. 66.0%, *P* = 0.003).

Following failure of first-line IST+EPAG, 7 patients underwent salvage HSCT, including 2 from matched sibling donors, 4 from matched unrelated donors, and 1 from a haploidentical donor. No patient in the HID-HSCT group required a second transplantation.

### Safety and complications

The safety profiles of the two treatment strategies differed (Table 3). While HID-HSCT demonstrated superior hematological response, it was associated with a higher incidence of infectious complications, particularly severe infections (49.3% vs. 25.0%, *P* = 0.035) and CMV viremia (55.2% vs. 19.6%, *P* < 0.001). In contrast, rates of severe hemorrhage, drug-related kidney injury, and EBV viremia were comparable between groups. Secondary clonal disease (*n* = 2) and serum sickness (*n* = 5) occurred exclusively in the IST+EPAG group, while TA-TMA (*n* = 6) occurred exclusively in the HID-HSCT group.

**Table 3:** Hematological response and complications of patients in the IST+EPAG and HID-HSCT groups

Efficacy	IST+EPAG *n* = 56 (%)	HID-HSCT *n* = 67 (%)	*P*
3-month response			< 0.001**
CR	3(5.4)	12(17.9)	
PR	18(32.1)	42(62.7)	
NR	32(57.1)	9(13.4)	
Death	3(5.4)	4(6.0)	
6-month response			< 0.001**
CR	10(17.9)	30(44.8)	
PR	20(35.7)	20(29.9)	
NR	21(37.5)	5(7.5)	
Death	5(8.9)	12(17.9)	
12-month response			< 0.001**
CR	19(33.9)	43(64.2)	
PR	18(32.1)	8(11.9)	
NR	12(21.4)	2(3.0)	
Death	7(12.5)	14(20.9)	
Severe infections	14(25.0)	33(49.3)	0.035*
Severe hemorrhage	4(7.1)	4(6.0)	0.646
Drug-related kidney injury	9(16.1)	7(10.4)	0.372
CMV viremia	11(19.6)	37(55.2)	< 0.001*
EBV viremia	7(12.5)	8(11.9)	0.316
Serum disease	5(8.9)	0(0.0)	-
Secondary clonal disease	2(3.6)	0(0.0)	-
TA-TMA	0(0.0)	6(9.0)	-

* P < 0.05. ** P < 0.01

*CR* complete response, *PR* partial response, *ORR* overall response rate, *NR* No response, *CMV* cytomegalovirus, *EBV* Epstein-Barr Virus, *TA-TMA* Transplant-associated thrombotic microangiopathy

Infection was the leading cause of death in both groups (HID-HSCT: *n* = 10; IST+EPAG: *n* = 9). Additional causes in the HID-HSCT group were intracranial hemorrhage (*n* = 2), TA-TMA (*n* = 2), aGVHD (*n* = 1), and hemophagocytic syndrome (*n* = 1). In the IST+EPAG group, deaths also resulted from leukemic transformation (*n* = 1) and intracranial hemorrhage (*n* = 1).

Overall, HID-HSCT offered superior and faster hematological response, but at the cost of increased infectious morbidity, highlighting the need for enhanced infection prophylaxis and monitoring in transplant recipients.

### Survival outcomes

**Fig. 4:**
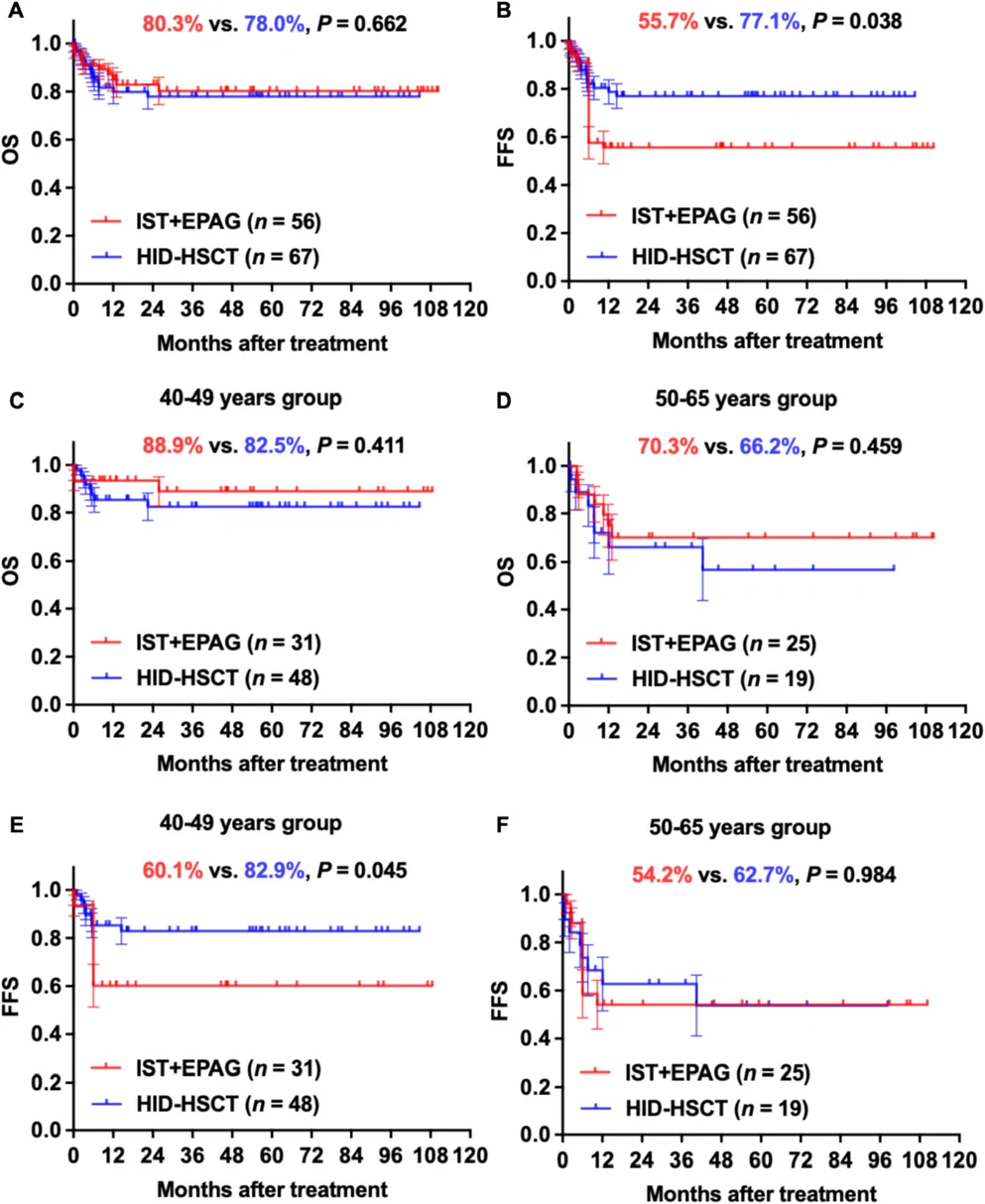
The assessed survival of the IST+EPAG and HID-HSCT groups (**A**) Overall survival (OS) at 3 years in the IST+EPAG and HID-HSCT groups. (**B**) Failure-free survival (FFS) at 3 years in the IST+EPAG and HID-HSCT groups. (**C**) OS at 3 years in patients age 40–49 years. (**D**) OS at 3 years in patients age 50–65 years. (**F**) FFS at 3 years in patients age 40–49 years. (**F**) FFS at 3 years in patients age 50–65 years

While 3-year OS was similar between the HID-HSCT and IST+EPAG groups (78.0 ± 5.2% vs. 80.3 ± 5.7%, *P* = 0.662; Fig. 4A), the HID-HSCT group achieved a significantly higher 3-year FFS rate (77.1 ± 5.2% vs. 55.7 ± 6.8%, *P* = 0.038; Fig. 4B). To account for the higher early mortality in the HID-HSCT arm, we performed a landmark analysis conditional on surviving to 6 months. After excluding patients who died before the 6-month landmark, the subsequent 3-year conditional OS was comparable between the two groups (HID-HSCT: 92.2% vs. IST+EPAG: 88.9%, *P* = 0.616, Supplementary Figure S1). This suggests that the survival disadvantage of HID-HSCT was largely confined to the early post-treatment period. We also evaluated GFFS to capture the morbidity burden of significant GVHD. The 3-year GFFS in the HID-HSCT group was 77.3 ± 5.2% (Supplementary Figure S2). For the IST+EPAG group, as no patients developed GVHD, the GFFS was equivalent to the FFS (55.7 ± 6.8%).

### Subgroup analyses by age and disease severity

Age-stratified subgroup analyses further elucidated these findings. OS remained comparable between the two treatment modalities across both age subgroups (40–49 years and 50–65 years; Fig. 4C and D). However, the FFS benefit associated with HID-HSCT was particularly pronounced in the younger cohort (aged 40–49 years), with rates of 82.9% ± 5.5% versus 60.1% ± 8.9% in the IST+EPAG group (P = 0.045; Fig. 4E). No significant FFS difference was observed among patients aged 50–65 years (*P* = 0.984; Fig. 4F). These results indicate that transplantation more effectively prevents treatment failure, including relapse and clonal evolution.

To evaluate whether the treatment benefit persisted in the most severe patients, we performed subgroup analyses stratified by disease severity (Supplementary Figure S3). Consistent with the main analysis, the 3-year FFS favored HID-HSCT (78.2% vs. 24.0%, *P* = 0.002) in the VSAA subgroup.

Multivariable Cox regression analysis adjusted for treatment group, age (50-65 vs. 40-49), and disease severity (VSAA vs. SAA) was performed (Table 4). FFS remained significantly superior in the HID-HSCT group (aHR = 0.496, 95% CI: 0.253–0.972, *P* = 0.041), indicating that the transplantation benefit is independent of age and severity. OS showed no significant difference between groups (aHR = 1.161, *P* = 0.729). As anticipated, VSAA was associated with significantly worse OS (aHR = 2.351, *P* = 0.042) and a trend toward worse FFS (aHR = 1.852, *P* = 0.066), confirming that disease severity is a valid prognostic factor in our cohort. These adjusted analyses support the robustness of our primary conclusion that HID-HSCT offers an FFS advantage over IST+EPAG, independent of baseline imbalances.

**Table 4:** Multivariable Cox regression analysis for OS and FFS

Variable	OS –aHR (95% CI)	*P*	FFS– aHR (95% CI)	*P*
Group (HID-HSCT vs. IST+EPAG)	1.161(0.500–2.698)	0.729	0.496 (0.253–0.972)	0.041
Age (50–65 vs. 40–49)	2.182(0.954–4.989)	0.064	1.525 (0.800–2.906)	0.200
Severity (VSAA vs. SAA)	2.351(1.030–5.369)	0.042	1.852(0.960–3.573)	0.066

*aHR* adjusted hazard ratio, *CI* confidence interval, *OS* overall survival, *FFS* failure-free survival, *VSAA* very severe aplastic anemia, *SAA* severe aplastic anemia

## Discussion

This study compared the efficacy and safety of first-line HID-HSCT and IST+EPAG in patients aged 40–65 years with SAA. Our results showed that HID-HSCT led to a faster and more robust hematological response than IST+EPAG, with significantly higher CR and ORR at 3, 6, and 12 months. Although 3-year OS did not differ significantly between groups, FFS was notably higher in the HID-HSCT arm among patients aged 40–49 (82.9% vs. 60.1%, *P* = 0.045), suggesting more durable disease control with transplantation in this subgroup. In contrast, no FFS benefit was observed for patients aged 50–65 (*P* = 0.984).

Our results align with recent evidence advocating for expanded HSCT eligibility in older adults with appropriate health status. Xu et al. reported 3-year OS of 77.2% in older SAA patients undergoing HSCT, consistent with our findings [27]. The data support the growing trend of using HID-HSCT for patients lacking an MSD or MUD, with outcomes approaching those of MSD-HSCT. Similarly, Liu et al. demonstrated superior FFS (77.8% vs. 48%, *P* < 0.0001) and better HRQoL with HID-HSCT versus IST in patients aged ≤ 55 years [6].

Currently, most centers still use 40 years as the cutoff age for first-line treatment of MSD-HSCT. While the cut-off age for MSD-HSCT appears to be shifting toward 50 years, there is no consensus yet [3, 27]. Our findings suggest that for patients aged 40–49 with good performance status, age alone should not preclude HID-HSCT, as the accelerated hematopoietic recovery and improved FFS may outweigh the risks in this subgroup. For older patients (50–65), careful patient selection remains critical given the lack of clear FFS benefit in this age range.

Despite the promising efficacy of IST combined with EPAG, this treatment approach remains limited by delayed response times. For SAA, response to ATG is delayed, starting after 3–4 months, and on average the 6-month response rate is 50%–70% [12]. The lower 6-month ORR in our IST+EPAG arm (53.6%) versus the RACE trial (63.6% in patients > 40 years) [2] may reflect differences in ATG type/dosing, variable EPAG exposure, racial variations in IST response, or modest sample size. Given this low response rate to second ATG, early transition to HSCT should be considered for medically fit patients, particularly those with refractory AA [12]. 

Our findings underscore a critical trade-off: HID-HSCT offers superior hematological recovery and FFS, but at the cost of heightened early mortality, predominantly from infections. The near-doubling of 6-month mortality (17.9% vs. 8.9%) reflects the well-recognized toxicity of haploidentical transplantation in SAA, including infection, TA-TMA and aGVHD. Notably, the survival disadvantage appears confined to the first 6 months—patients who survive this period achieve durable outcomes comparable to IST+EPAG. For older patients or those with significant comorbidities, however, this early risk may be unacceptable, emphasizing the need for meticulous pre-transplant risk assessment, infection prophylaxis, and aggressive supportive care.

Our 100-day II-IV aGVHD rate (30.9%) exceeded that reported by DeZern et al. (11% in a 37-patient SAA series) [28], likely reflecting our older age and heterogeneous conditioning. Our 3-year cGVHD rate (8.1%) was comparable to their 2-year cGVHD rate (8%). These comparisons underscore the impact of protocol standardization on GVHD outcomes [29]. 

This study has several limitations. First, as a non‑randomized cohort study, selection bias cannot be excluded—baseline imbalances in VSAA (41.8% vs. 26.8%, *P* = 0.127) and age 50–65 (28.4% vs. 44.6%, *P* = 0.275) reflect real‑world preferences but were addressed by multivariable adjustment **(**Table 4**)**. Second, the modest sample size precluded propensity‑score matching and constrained multivariable modeling, so residual confounding may persist. Third, HRQoL was not assessed, and follow‑up, while adequate for 3‑year outcomes, may miss secondary malignancies or late organ dysfunction. Fourth, the 8‑year study period (2016–2024) spans significant practice shifts—EPAG adoption and the transition from BuCy to PTCy‑based conditioning after 2018—meaning the IST+EPAG arm does not uniformly reflect the current post‑RACE standard throughout. Finally, our findings require validation in larger multicenter studies with propensity-matched designs and different transplantation protocols.

Despite these limitations, this study provides valuable real-world evidence that first-line HID-HSCT is a compelling alternative to IST+EPAG for carefully selected patients aged 40–49 years with suitable donors and good performance status, particularly when rapid, complete hematologic recovery and prevention of treatment failure are prioritized. Future prospective studies with larger samples and longer follow-up are needed to refine selection criteria and optimize outcomes in this understudied population.

## Supplementary information

Below is the link to the electronic supplementary material.

Supplementary Material 1

## Data Availability

The data that support the findings of this study are available from the corresponding author upon reasonable request.
