# Supplementary material for: Haploidentical hematopoietic stem cell transplantation compared to immunosuppressive therapy plus eltrombopag as first-line treatment for severe aplastic anemia patients aged 40–65: a multicenter retrospective study
Source: Ann Hematol. 2026 Sep 29;105(11):468. doi: 10.1007/s00277-026-07289-2 (PMC13683484; doi:10.1007/s00277-026-07289-2)
Supplement: Supplementary file 1 [file 277_2026_7289_MOESM1_ESM.docx]

**Supplementary Material**

**Haploidentical hematopoietic stem cell transplantation compared to immunosuppressive therapy plus eltrombopag** **as first-line treatment for severe aplastic anemia patients aged 40–65: a multicenter retrospective study**

Xiaohui Xiao,^1,2*^ Guiping Chen,^2,3*^ Cong Xu,^1,*^ Liangliang Wu,^2,3^ Xiaoyan Liu^4,5^, Ming Zhou,^2,3^ Xiaowei Chen,^2,3^ Ruiqing Zhou,^2,3^ Wenjian Mo,^2,3^ Yumiao Li,^2,3^ Shilin Xu,^2,3^ Yuping Zhang,^2,3,#^ Dongjun Lin,^1,#^ and Shunqing Wang^2,3,#^

^1^Department of Hematology, The Seventh Affiliated Hospital, Sun Yat-Sen University, Shenzhen, China;

^2^Department of Hematology, Guangzhou First People's Hospital, Institute of Blood Transfusion and Hematology, Guangzhou Medical University, Guangzhou, China;

^3^Department of Hematology, Guangzhou First People's Hospital, South China University of Technology, Guangzhou, China;

^4^Department of Hematology, Sun Yat-sen Memorial Hospital, Sun Yat-sen University, Guangzhou, China;

^5^Guangdong Provincial Key Laboratory of Malignant Tumor Epigenetics and Gene Regulation, Sun Yat-Sen Memorial Hospital, Sun Yat-Sen University, Guangzhou, China

**Landmark analysis**

To separate early transplant-related mortality from late outcomes, a landmark analysis was performed at 6 months post-treatment. Patients who died or were lost to follow-up before the 6-month landmark were excluded. The conditional overall survival from the 6-month landmark to the end of follow-up was estimated using Kaplan-Meier method and compared between groups by log-rank test.

**Supplementary Figure S1. Landmark analysis of overall survival from 6 months post-treatment, conditional on surviving to 6 months.**

**Supplementary Figure S2. Kaplan-Meier curves for GVHD-free failure-free survival (GFFS) in HID-HSCT patients.**

**Supplementary Figure S3. Subgroup survival analyses stratified by disease severity**

(A) OS at 3 years in patients with VSAA. (B) OS at 3 years in patients with SAA. (C) FFS at 3 years in patients with VSAA. (D) FFS at 3 years in patients with SAA.

Abbreviations: OS, overall survival; FFS, failure-free survival; VSAA, very severe aplastic anemia; SAA, severe aplastic anemia.
